# Sensitivity of point-of-care IgM and IgG test in critically ill patients with SARS-Cov-2

**DOI:** 10.1186/s13054-020-03290-x

**Published:** 2020-09-24

**Authors:** Lee S. Nguyen, Driss Laghlam, Emmanuelle De Gonfreville, Frédéric Pène, Flore Rozenberg, Jean-Paul Mira

**Affiliations:** 1 Intensive Care Medicine Department, AP.HP.Centre Cochin University Hospital, Paris, France; 2 Intensive Care Medicine Department, CMC Ambroise Paré, Neuilly-sur-Seine, France; 3 Virology Department, AP.HP.Centre Cochin University Hospital, Paris, France

Dear Editor,

The severe acute respiratory syndrome coronavirus 2 (SARS-Cov-2) pandemic requires accurate diagnostic tests to triage patients between properly isolated and regular wards [1]. Gold-standard tests are based on reverse-transcriptase polymerase chain reaction (RT-PCR) performed on nasopharyngeal swabs [2]. Quick serology tests detecting immunoglobulin M and G (respectively IgM and IgG) targeted against SARS-Cov-2 are also available [3, 4]; however, concerns have been raised on their sensitivity in intensive care units (ICU), where patients are more severe and some immunosuppressed.

In this multicenter observational study, we assessed sensitivity of a point-of-care serology test (POCST) regarding SARS-Cov-2, in ICU patients presenting severe SARS-Cov-2 infection. All included patients were positive for SARS-Cov-2 using routine RT-PCR methodology. POCST was sampled with finger prick, with 10 μL of blood and tested with the device, BIOSYNEX COVID-19 BSS (IgG/IgM)® (*Biosynex,* Illkirch-Graffenstaden, France). Each POCST incorporated a quality control. Concordance between RT-PCR and POCST was assessed regarding the presence of IgM and/or IgG. Patients for whom POCST showed no IgM and no IgG were considered negative. The study was approved by institutional review board (00012608-2020-01) and registered under clinicaltrials.gov identifier NCT04467008.

Overall, 99 patients were included. Patients were 62.4 ± 13.3 years old, 34.7% were women, and average body-mass index (BMI) was 29.1 ± 5.9 kg/m^2^. Mean Simplified Acute Physiological Score II (SAPS II) was 50.1 ± 22.8. Average delay between POCST and first symptoms was 17.9 ± 9.1 days (see baseline characteristics in Table 1). Results were obtained in less than 10 min for all, except in 2 (2.0%) in whom quality control was not met; hence, tests required to be performed twice.

**Table 1:** Study cohort baseline characteristics (all patients had confirmed COVID-19 pneumonia)

	Overall (*n* = 99)	Positives (*n* = 91)	Negatives (*n* = 8)	Intergroup comparison *p* value
Age (years)	62.4 ± 13.3	63.5 ± 12.5	50.7 ± 16.9	0.009^ǂ^
Woman patient	34 (34.3%)	32 (35.2%)	2 (25.0%)	0.71^Φ^
Body-mass index (kg/m^2^)	29.1 ± 5.9	29.2 ± 5.8	29.1 ± 7.1	0.94^Μ^
Delay between first symptoms and POCST (days)	17.9 ± 8.2	18.6 ± 7.9	10.4 ± 7.8	0.006^ǂ^
Chronic immunosuppression	9 (9.1%)	7 (7.7%)	2 (25.0%)	0.15^Φ^
Diabetes	31 (31.3%)	28 (30.8%)	3 (37.5%)	0.70^Φ^
Corticosteroid use in the past 14 days	18 (18.2%)	16 (17.6%)	2 (25.0%)	0.63^Φ^
Immunosuppression in the past 14 days	5 (5.1%)	5 (5.5%)	0 (0.0%)	1.0^Φ^
SAPS II at admission	50.1 ± 23.0	51.1 ± 22.4	40.4 ± 28.7	0.17^Μ^
Creatininemia at admission (μmol/L)	106.4 ± 123.7	106.4 ± 128.6	107.3 ± 45.8	0.047^Μ^
Lymphocytes’ count on day of POCST (G/L)	2.3 ± 8.2	2.4 ± 8.5	1.0 ± 0.7	0.56^Μ^
Fibrinogen on day of POCST (g/L)	5.8 ± 2.8	5.8 ± 2.8	4.4 ± 1.2	0.11^Μ^

Chronic immunosuppression denotes either human immunodeficiency virus, solid organ transplantation or allogeneic hematopoietic stem cell transplantation

*POCST* point-of-care serology test, *SAPS II* Simplified Acute Physiological Score II

^Μ^Mann-Whitney *U* test for distribution

^ǂ^Student’s *t* test

^Φ^Fischer’s exact test

The POCST yielded 8 (8.1%) negatives, corresponding to a sensitivity of 91.9%. Delay between first symptoms and POCST was significantly lower in negative than positive patients (10.4 ± 7.8 vs 18.6 ± 7.9 days, *p* = 0.005) (see Fig. 1 a). Negatives were significantly younger (50.7 ± 16.9 vs. 63.5 ± 12.5 years old, *p* = 0.009). Rest of variables were similar, including lymphocytes’ count (1.0 ± 0.7 vs 2.4 ± 8.5 G/L, *p* = 0.55) (see Table 1 and Fig. 1). Multivariable logistic regression showed that both delay and age were independently associated with negative POCST (respectively adjusted odds-ratio, 0.82 (0.71–0.95) per 1-day increase, *p* value = 0.009, and 0.93 (0.87–0.98) per 1-year increase, *p* value = 0.013).

**Fig. 1:**
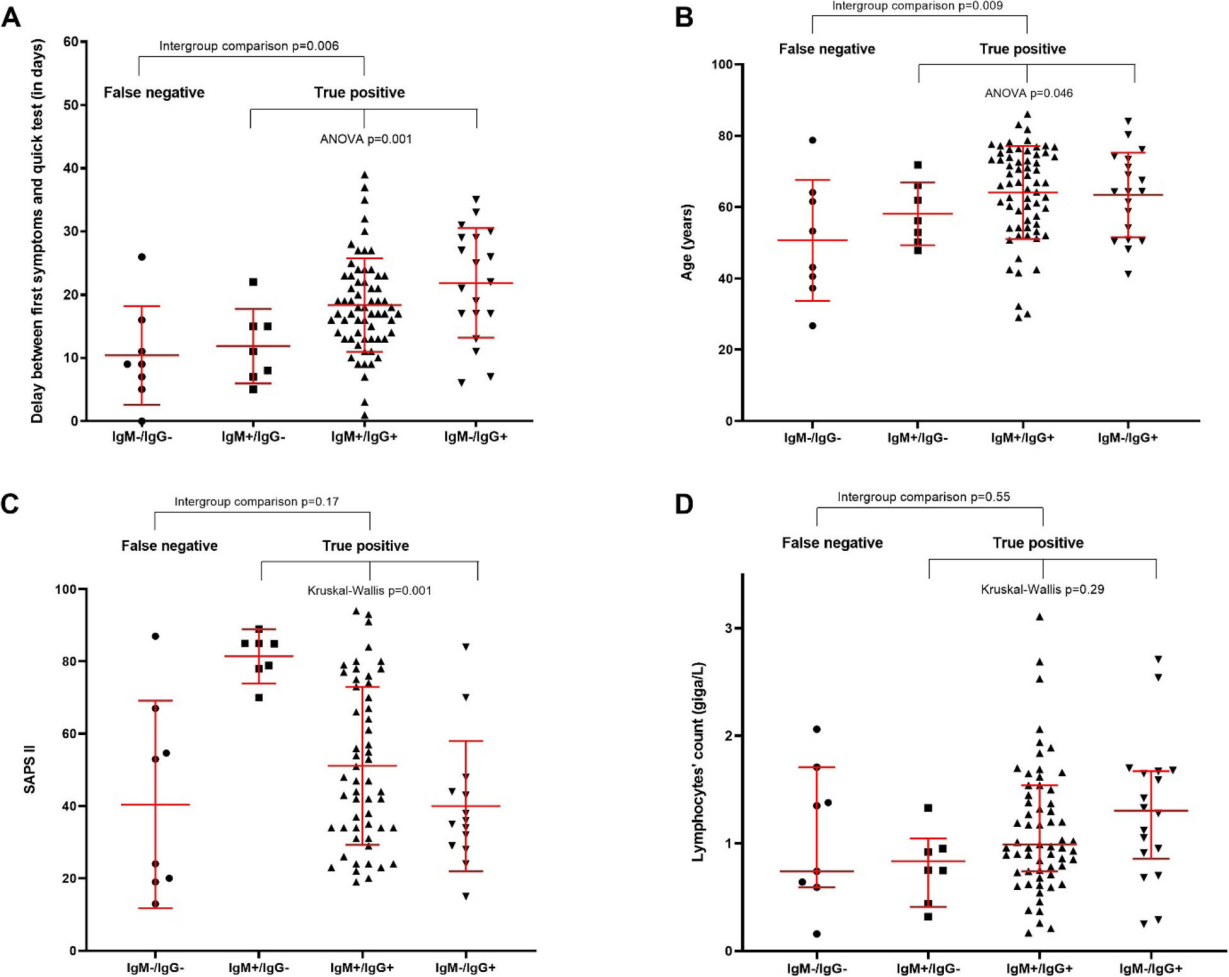
Delay between first symptoms of SARS-Cov-2 and quick serology test (in **a**), age (in **b**), Simplified Acute Physiological Score II (SAPS II, in **c**), and lymphocytes’ count on the day of quick serology test (in **d**). In **a** and **b**, red whiskers represent mean and standard deviation; in **c** and **d**, they represent median and interquartile range, due to non-Gaussian distribution. Comparisons between false-negative and true-positive test results were performed using *t*-test for delay and age, and Mann-Whitney test for SAPS II and lymphocytes’ count. Four-group comparisons were performed using analysis of variance (ANOVA) for delay and age, and Kruskal-Wallis test for SAPS II and lymphocytes’ count

The other three different serology profiles were IgM+/IgG− in 7, IgM+/IgG+ in 64, and IgM−/IgG+ in 19 patients. Delay between first symptoms and POCST was significantly different across all four groups. Contrary to SAPS II, distribution of age, BMI, and lymphocytes’ count did not significantly differ across all four groups (see Fig. 1b–d).

In this observational study in ICU patients, sensitivity of POCST was similar to specifications provided by the manufacturer (93%). Variables associated with negative results were age and delay between onset and POCST which was expected given known dynamics of immunization after SARS-Cov-2 infection [5]. Neither patients’ severity nor immunosuppression status modified risk of presenting negative POCST results. Lymphocytes’ count was not significantly different, however; it was twice as lower in false-negative patients although a lack of power be incriminated.

Although multicenter, this study suffers from small sample size, hence validation in larger cohorts aiming at assessing effects of POCST on in-hospital virus contamination and beds management may answer whether these quick diagnostic tests alleviate burden of SARS-Cov-2 on ICU beds and staff [6].

To conclude, we assessed diagnostic performance of a point-of-care serology test for SARS-CoV-2 in 99 patients hospitalized in ICU with a definite SARS-Cov-2 and found a 91.9% sensitivity, confounded by younger age and shorter delay since symptoms onset. POCST sensitivity was not considered elevated enough in clinical practice to help triage between SARS-CoV-2 isolated wards and regular ICU wards.

## Data Availability

The datasets used and/or analyzed during the current study are available from the corresponding author on reasonable request.
